# Supplementary material for: Flocculant-Assisted Synthesis of Graphene-Like Carbon Nanosheets for Oxygen Reduction Reaction and Supercapacitor
Source: Nanomaterials (Basel). 2019 Aug 7;9(8):1135. doi: 10.3390/nano9081135 (PMC6722949; doi:10.3390/nano9081135)
Supplement: Supplementary file 1 [file nanomaterials-09-01135-s001.pdf]

Supplementary Information

Flocculant-Assisted Synthesis of Graphene-Like Carbon Nanosheets for Oxygen Reduction Reaction and Supercapacitor

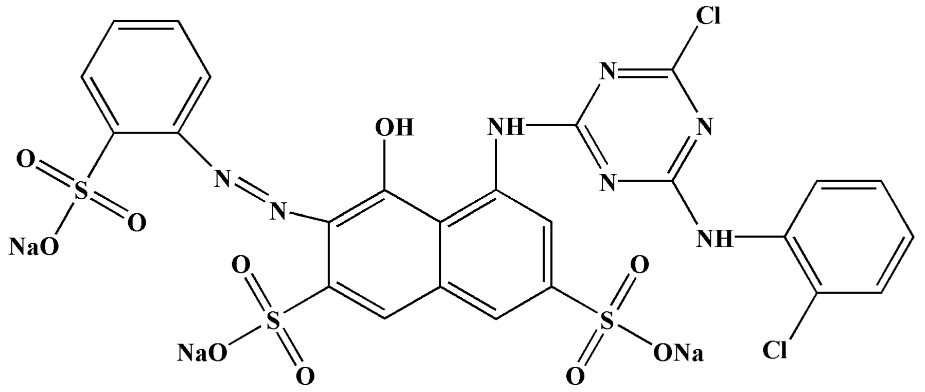

Figure S1. The structure of K-2BP.

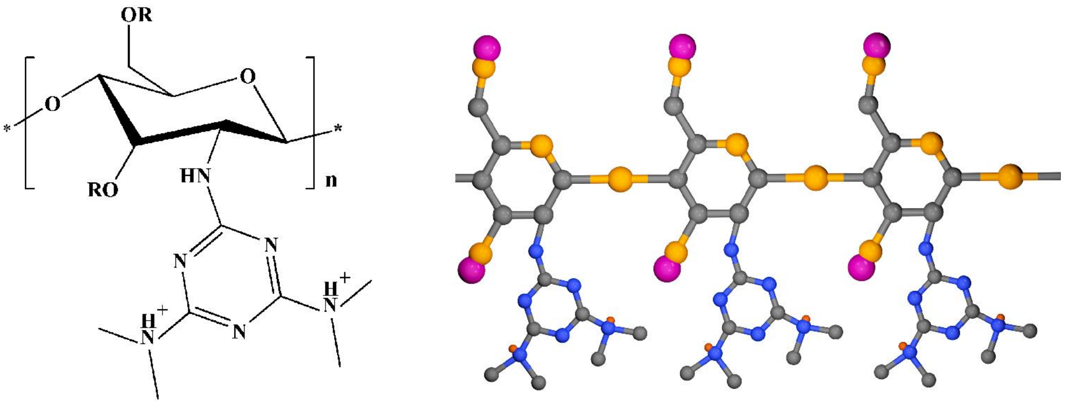

Figure S2. The structure of 2,4-bis(dimethyl amino)-6-chloro-[1,3,5]-triazine-chitosan (BDAT-CTS).

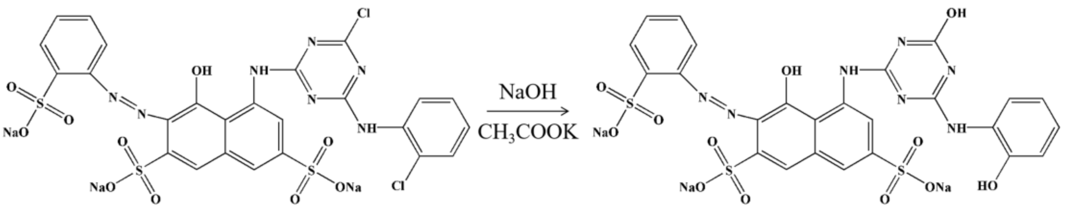

Figure S3. The equation to hydrolysis of K-2BP.

Table S1. The textural parameters of NSC-Fe-*x* materials.

Samples	NSC-Fe-1	NSC-Fe-2	NSC-Fe-3
S_{BET} ($m^2 g^{-1}$)	368.17	499.60	466.80
S_{micro} ($m^2 g^{-1}$)	296.70	479.65	382.75
S_{meso} ($m^2 g^{-1}$)	71.47	19.95	84.05
d_{HK} (nm)	0.40	0.36	0.41
d_{BJH} (nm)	3.80	3.73	3.39
V_{total} (cm^3g^{-1})	0.39	0.26	0.30
V_{micro} (cm^3g^{-1})	0.14	0.22	0.18
V_{meso} (cm^3g^{-1})	0.25	0.04	0.12

HK: Horvaih–Kawazoe. BJH: Barrett–Joyner–Halenda.

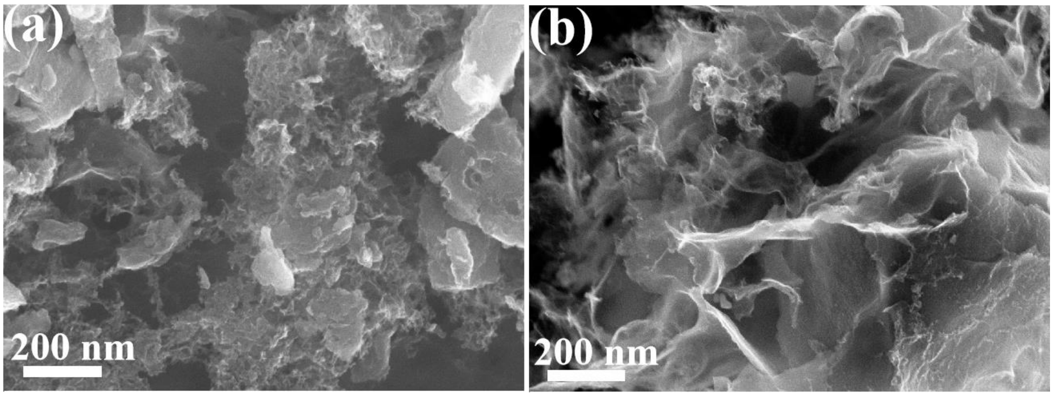

Figure S4. SEM images of (**a**) NSC-Fe-1 and (**b**) NSC-Fe-3.

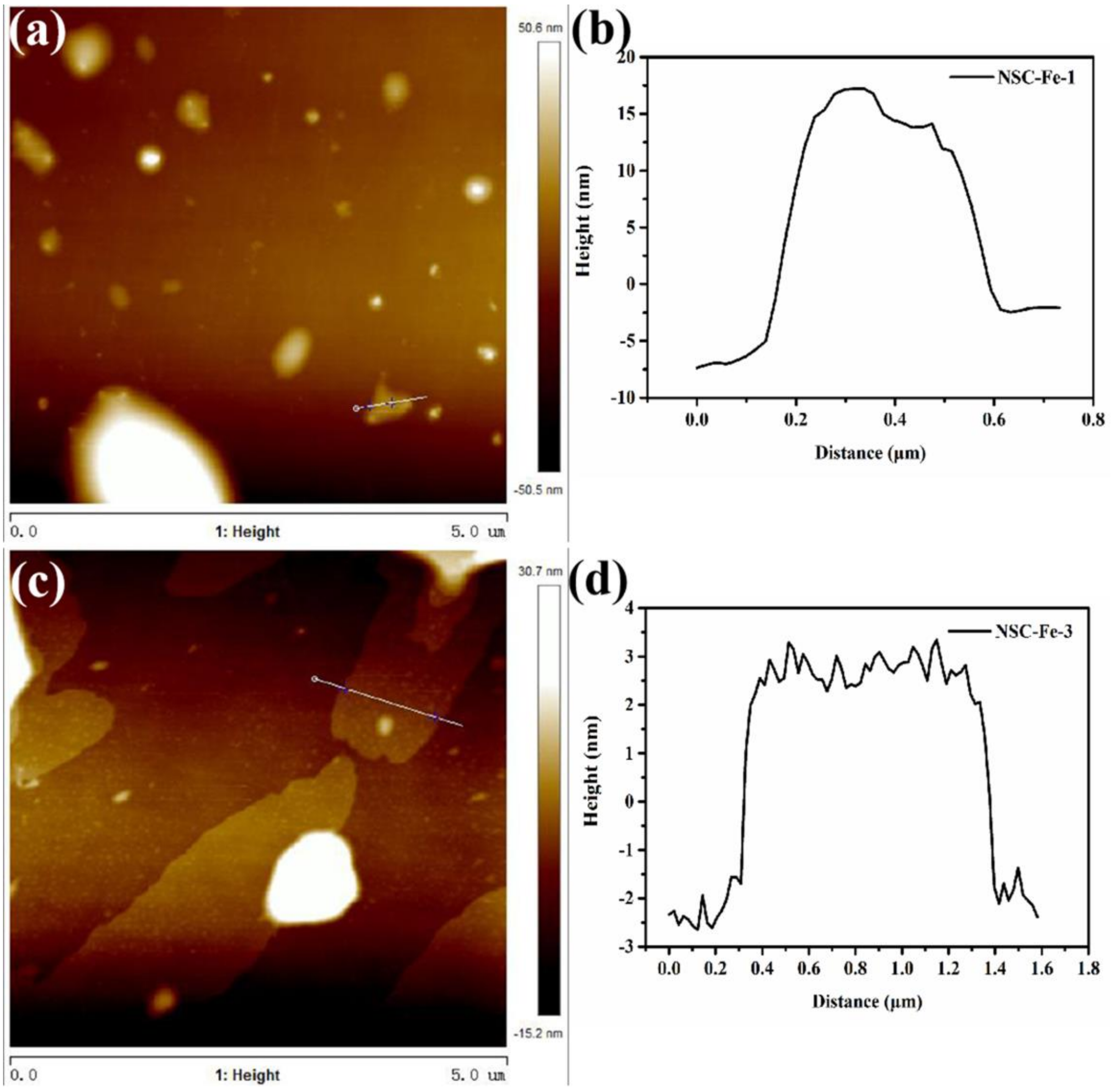

Figure S5. AFM images and the thickness of NSC-Fe-1 (**a**, **b**) and NSC-Fe-3 (**c**, **d**).

Table S2. The concentrations of N species in NSC-Fe-x catalysts determined by XPS measurements.

N1s	Pyridinic-N	Pyrrolic-N	Graphitic-N	Oxide-N
NSC-Fe-1	28.92%	60.57%	5.26%	5.25%
NSC-Fe-2	23.37%	40.46%	29.22%	6.95%
NSC-Fe-3	29.36%	41.83%	22.44%	6.37%

Table S3. Comparation of NSC-Fe-2 with carbon materials for oxygen reduction reaction (ORR) reported recently. (In the table, reversible hydrogen electrode was abbreviated as RHE).

Catalysts	Raw Materials	Onset Potential (V vs. RHE)	Stability Result (%)	Methanol Tolerance Result (%)	Ref.
Fe-S-N HPCNR	Cysteine, Fe^{2+} salt, ZnO NRs@PDA	0.93	92 (20000 s)	~ 100	1
$Fe/Fe_2N/Fe_3CNP$	Fructose, $FeCl_3$	1.03	83 (12 h)	~ 100	2
Fe-N-C/rGO	Acetone, reduced graphene oxides, ammonium persulfate	0.94	92.8 (12000 s)	> Pt/C	3
$Fe_3O_4/Fe/C$	$Fe(NO_3)_3.9H_2O$, H_4BTA, dimethylformamide	0.87	67 (28800 s)	> Pt/C	4
FeN_x/C	α-Fe_2O_3, agarose	1.1	90 (20000 s)	> Pt/C	5
FeS/Fe_3C@N-S-C	3,8-dibromophenanthroline, 1, 3, 5 benzenetriboronicacid trivalent alcohol ester, Iron (II) acetate	0.87	~	~	6
Fe–N–C HNS	Histidine, SiO_2-templated	1.046	91 (20000 s)	~ 100	7
Fe/N/S-PCNT	$FeCl_3$, pyrrole monomer	0.96	96 (30000 s)	~ 100	8
NSC-Fe-2	Dye wastewater, chitosan, $Fe_2(SO_4)_3$	1.05	88.3 (33300 s)	98 (3 M)	This work

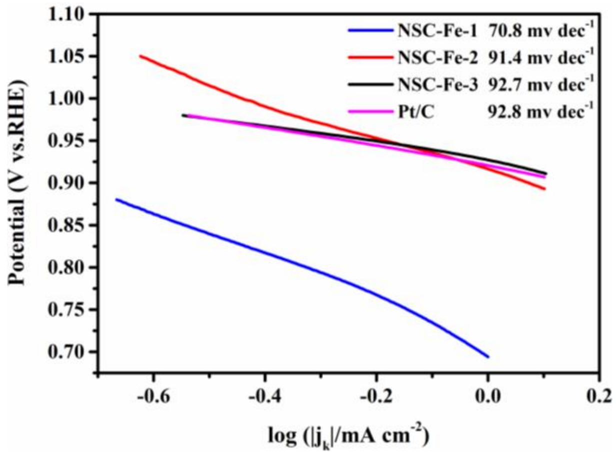

Figure S6. Tafel plots of NSC-Fe-1, NSC-Fe-2, NSC-Fe-3 and commercial Pt/C catalysts.

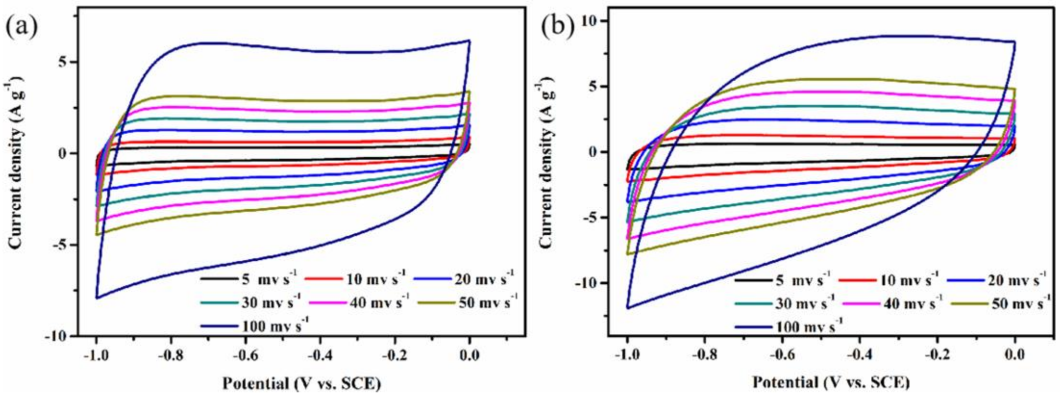

Figure S7. CV curves at different scan rates of (a) NSC-Fe-1, and (b) NSC-Fe-3.

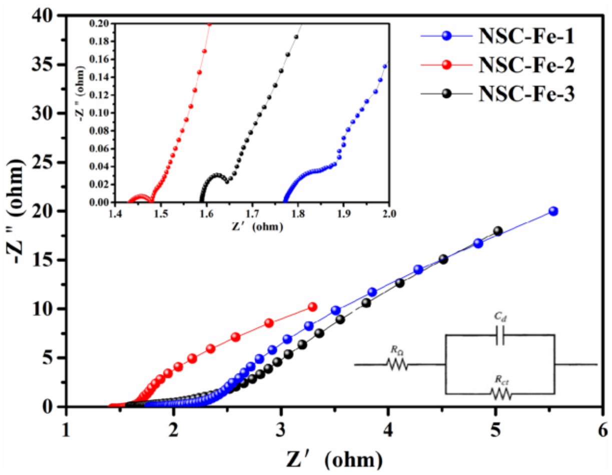

Figure S8. Nyquist plot of the EIS for NSC-Fe-1, NSC-Fe-2 and NSC-Fe-3.

References

1. Wang, Y.; Zhu, C.; Feng, S.; Shi, Q.; Fu, S.; Du, D.; Zhang, Q.; Lin, Y., Interconnected Fe, S, N-Codoped Hollow and Porous Carbon Nanorods as Efficient Electrocatalysts for the Oxygen Reduction Reaction. *ACS Appl. Mater. Interfaces* **2017**, *9*, 40298-40306.
2. Cao, L.; Li, Z.-h.; Gu, Y.; Li, D.-h.; Su, K.-m.; Yang, D.-j.; Cheng, B.-w., Rational design of N-doped carbon nanobox-supported Fe/Fe_2N/Fe_3C nanoparticles as efficient oxygen reduction catalysts for Zn–air batteries. *J. Mater. Chem. A* **2017**, *5*, 11340-11347.
3. Zhang, C.; Liu, J.; Ye, Y.; Aslam, Z.; Brydson, R.; Liang, C., Fe-N-Doped Mesoporous Carbon with Dual Active Sites Loaded on Reduced Graphene Oxides for Efficient Oxygen Reduction Catalysts. *ACS Appl. Mater. Interfaces* **2018**, *10*, 2423-2429.
4. Devi, B.; Venkateswarulu, M.; Kushwaha, H. S.; Halder, A.; Koner, R. R., A Polycarboxyl-Decorated Fe(III) -Based Xerogel-Derived Multifunctional Composite (Fe_3O_4/Fe/C) as an Efficient Electrode Material towards Oxygen Reduction Reaction and Supercapacitor Application. *Chem. Eur. J.* **2018**, *24*, 6586-6594.
5. Han, S.; Hu, X.; Wang, J.; Fang, X.; Zhu, Y., Novel Route to Fe-Based Cathode as an Efficient Bifunctional Catalysts for Rechargeable Zn-Air Battery. *Adv. Energy Mater.* **2018**, *8*, 1800955.
6. Kong, F.; Fan, X.; Kong, A.; Zhou, Z.; Zhang, X.; Shan, Y., Covalent Phenanthroline Framework Derived FeS@Fe_3C Composite Nanoparticles Embedding in N-S-Codoped Carbons as Highly Efficient Trifunctional Electrocatalysts. *Adv. Funct. Mater.* **2018**, *28*, 1803973.
7. Chen, Y.; Li, Z.; Zhu, Y.; Sun, D.; Liu, X.; Xu, L.; Tang, Y., Atomic Fe Dispersed on N-Doped Carbon Hollow Nanospheres for High-Efficiency Electrocatalytic Oxygen Reduction. *Adv. Mater.* **2019**, *31*, 1806312.
8. Tan, Z.; Li, H.; Feng, Q.; Jiang, L.; Pan, H.; Huang, Z.; Zhou, Q.; Zhou, H.; Ma, S.; Kuang, Y., One-pot synthesis of Fe/N/S-doped porous carbon nanotubes for efficient oxygen reduction reaction. *J. Mater. Chem. A* **2019**, *7*, 1607-1615.
